# Comparison of lamina cribrosa depth shallowing after trabeculectomy between primary open-angle glaucoma and exfoliation glaucoma

**DOI:** 10.1038/s41598-022-19785-2

**Published:** 2022-09-20

**Authors:** Do Young Park, Sunggeun Son, Soon Cheol Cha

**Affiliations:** Department of Ophthalmology, Yeungnam University Hospital, Yeungnam University College of Medicine, Daegu, Korea

**Keywords:** Optic nerve diseases, Glaucoma

## Abstract

The lamina cribrosa (LC) becomes shallower as intraocular pressure (IOP) decreases after trabeculectomy. The LC in eyes with exfoliation syndrome has distinctive properties in the connective tissue and extracellular matrix, but how these affect the changes in LC depth in response to IOP reduction after trabeculectomy is unknown. We analyzed pre- and postoperative spectral-domain optical coherence tomography of exfoliation glaucoma (XFG) and primary open-angle glaucoma (POAG) patients who underwent trabeculectomy and investigated whether LC depth differed between XFG and POAG eyes after trabeculectomy. In total, 30 XFG eyes and 30 visual field mean deviation-matched POAG eyes were included. LC depth was determined at an average of 3.9 months after trabeculectomy. Postoperatively, the LC depth became shallower and the BMO-MRW became thicker in both XFG and POAG eyes. XFG eyes showed lesser amount of LC depth shallowing than POAG eyes. Greater preoperative LC depth, lower postoperative IOP, and absence of XFG were all associated with a greater degree of postoperative LC depth shallowing. These findings suggest that the LC of XFG eyes may inherently possess the distinctive properties of the connective tissue and extracellular matrix contained within it, which could affect the LC response to the reduction in IOP after trabeculectomy.

## Introduction

Glaucomatous optic nerve head (ONH) changes include neuroretinal rim (NRR) thinning and deformation of the lamina cribrosa (LC)^1,2^. The NRR and LC are glial cell-based structures supporting axons of retinal ganglion cells (RGCs) at the level of the ONH and contain a fair amount of extracellular matrix (ECM)^1,2^. Thus, characteristics of glial cells and the ECM may affect glaucomatous ONH changes. These glaucomatous changes in NRR and the LC occur with increasing intraocular pressure (IOP), but reversible changes may also occur as the IOP decreases after glaucoma filtration surgery^3–6^. Specifically, it has been reported that the LC depth becomes shallower, the LC becomes thicker and flatter, and NRR thickness increases in response to decreased IOP after trabeculectomy^3–7^. These changes were reported to be related to the degree of IOP reduction after glaucoma surgery and the patient’s age, which are thought to be because the glial cells, ECM, and connective tissue, which consist of the NRR or LC, respond sensitively to changes in IOP^3–7^.

Exfoliation glaucoma (XFG) develops in patients with exfoliation syndrome (XFS), which is characterized by abnormal production and accumulation of fibrillary materials in various ocular tissues^8,9^. Besides the characteristic exfoliation materials observed at the pupil margin and lens capsules, the ONH in eyes with XFG is known to have different connective tissue and ECM properties of the LC^10^, which may determine the features of NRR thinning and LC deformation. The ONH in patients with XFG has been reported to have a thinner LC and steeper LC curvature compared to primary open-angle glaucoma (POAG) with the same degree of visual field (VF) damage^11,12^. For the NRR, there is disagreement among studies on what differences exist between eyes with POAG and XFG^13–15^. Some studies have shown that the NRR area of the ONH in eyes with XFG was smaller than that in POAG, while there was no significant difference between XFG and POAG in other studies^13,15^. However, to the best of our knowledge, reversal of the changes in the ONH in terms of LC deformation or NRR thinning in response to IOP reduction after glaucoma filtration surgery has not been studied yet.

We hypothesized that due to the different properties of connective tissue and ECM in the ONH in eyes with XFG, reversal of the changes in the ONH in terms of NRR thinning and LC deformation in response to decreased IOP after glaucoma filtration surgery could be different between XFG and POAG. To investigate this, we analyzed the postoperative ONH structural changes using LC parameters and Bruch’s membrane opening (BMO)-related parameters measured using optical coherence tomography (OCT) in glaucoma severity-matched POAG and XFG patients who underwent trabeculectomy.

## Results

In total, 30 consecutive eyes of 30 XFG patients and 30 VF MD-matched eyes of 30 POAG patients satisfying the inclusion criteria were included in the analysis. Intraobserver and interobserver intraclass correlation coefficients (ICCs) showed good agreement in LC depth measurements at all three locations (Supplementary Table 1). Demographic and baseline characteristics of the patients with POAG and XFG are summarized in Table 1. The mean age of POAG and XFG patients was 70.5 ± 6.6 years and 73.3 ± 5.6 years, respectively. There was no significant difference in axial length (AXL), central corneal thickness (CCT), VF parameters, preoperative LC depth, preoperative Bruch’s membrane opening minimum rim width (BMO-MRW), or preoperative retinal nerve fiber layer (RNFL) thickness between the two groups. The pre- and postoperative IOP were not significantly different between POAG eyes and XFG eyes (27.7 mmHg in POAG eyes and 28.0 mmHg in XFG eyes preoperatively, 10.6 mmHg in POAG eyes and 10.7 mmHg in XFG eyes postoperatively) (Table 1). Pre- and postoperative LC depth difference was significantly lower in eyes with XFG than those with POAG (POAG: 59.0 ± 56.2 μm vs XFG: 24.5 ± 39.7 μm, Table 1). The postoperative IOP decreased significantly in both groups compared to the preoperative IOP: from 27.7 mmHg preoperatively to 10.6 mmHg postoperatively in the POAG eyes and from 28.0 mmHg preoperatively to 10.7 mmHg postoperatively in the XFG eyes (Table 2).

**Table 1:** Demographic and baseline characteristics of patients with primary open-angle glaucoma and exfoliation glaucoma.

	POAG (n = 30)	XFG (n = 30)	p value
Age, years	70.5 ± 6.6	73.3 ± 5.6	0.085
Gender, male/female	19/11	21/9	0.784
Diabetes, yes/no	6/24	4/26	0.729
Hypertension, yes/no	5/25	9/21	0.360
Axial length, mm	24.4 ± 1.5	24.1 ± 0.8	0.358
Central corneal thickness, μm	551.9 ± 40.4	541.4 ± 28.1	0.248
Vertical cup to disc ratio	0.85 ± 0.09	0.88 ± 0.07	0.280
Disc size, μm^2^	2.2 ± 0.4	2.1 ± 0.3	0.278
Visual field MD, dB	− 20.2 ± 7.2	− 21.2 ± 6.6	0.570
Visual field PSD, dB	8.6 ± 2.7	7.5 ± 3.2	0.182
Phakia/pseudophakia	18/12	16/14	0.794
Duration, months	3.9 ± 1.0	3.9 ± 1.2	0.953
preoperative IOP, mmHg	27.7 ± 5.2	28.0 ± 7.7	0.876
postoperative IOP, mmHg	10.6 ± 3.1	10.7 ± 2.7	0.860
IOP Reduction, mmHg	17.2 ± 7.2	17.3 ± 7.8	0.945
Preoperative LC depth, μm	545.3 ± 184.0	538.9 ± 134.2	0.878
Postoperative LC depth, μm	486.3 ± 149.6	514.4 ± 123.8	0.539
Pre- and postoperative LC depth difference, μm	59.0 ± 56.2	24.5 ± 39.7	**0.008**
Preoperative BMO-area, μm^2^	2.4 ± 0.6	2.2 ± 0.4	0.189
Preoperatvie global BMO-MRW, μm	157.1 ± 43.7	140.1 ± 41.7	0.138
Postoperatvie global BMO-MRW, μm	193.9 ± 96.6	151.2 ± 44.1	**0.037**
Pre- and postoperative BMO-MRW difference, μm	35.6 ± 74.3	15.4 ± 41.0	0.200
Preoperatvie average RNFL thickness, μm	66.1 ± 20.5	58.0 ± 21.4	0.146
Postoperatvie average RNFL thickness, μm	65.0 ± 19.5	59.5 ± 19.8	0.294
Pre- and postoperative RNFL difference, μm	− 1.1 ± 11.0	1.5 ± 6.9	0.283

*POAG* primary open-angle glaucoma, *XFG* exfoliation glaucoma, *MD* mean deviation, *PSD* pattern standard deviation, *dB* decibel, *IOP* intraocular pressure, *LC* lamina cribrosa, *BMO-MRW* Bruch’s membrane opening-minimum rim width, *RNFL* retinal nerve fiber layer. Data are presented as mean ± standard deviation or n (frequency). Statistically significant p-values are shown in bold.

**Table 2:** Intraocular pressure, lamina cribrosa depth, Bruch’s membrane opening minimum rim width, and retinal nerve fiber layer thickness at preoperative and postoperative optical coherence tomography examination.

	POAG (n = 30)			XFG (n = 30)		
	Preoperative	Postoperative	p value	Preoperative	Postoperative	p value
IOP, mmHg	27.7 ± 5.2	10.6 ± 3.1	**< 0.001**	28.0 ± 7.7	10.7 ± 2.7	**< 0.001**
LCD 0°, μm	564.5 ± 192.7	509.3 ± 155.2	**< 0.001**	518.9 ± 140.9	489.5 ± 127.4	**0.002**
LCD 60°, μm	533.1 ± 181.2	477.8 ± 151.1	**< 0.001**	471.8 ± 147.6	448.1 ± 132.0	**0.002**
LCD 120°, μm	551.7 ± 185.7	481.4 ± 152.7	**< 0.001**	471.1 ± 127.5	452.0 ± 128.1	**0.040**
Average LCD, μm	545.3 ± 184.0	486.3 ± 149.6	**< 0.001**	488.9 ± 134.2	464.4 ± 123.8	**0.001**
BMO_area, μm^2^	2.4 ± 0.6	2.4 ± 0.6	0.784	2.2 ± 0.4	2.2 ± 0.4	0.837
BMO-MRW_G, μm	157.1 ± 43.7	193.9 ± 96.6	**0.003**	140.1 ± 41.7	151.2 ± 44.1	**0.005**
BMO-MRW _TS, μm	133.8 ± 57.8	152.3 ± 91.3	**0.028**	114.9 ± 46.4	123.0 ± 53.0	**0.046**
BMO-MRW _T, μm	137.4 ± 43.0	164.6 ± 85.5	**0.008**	119.3 ± 47.6	126.0 ± 46.7	0.054
BMO-MRW _TI, μm	158.9 ± 82.4	178.5 ± 123.7	0.125	116.6 ± 68.9	125.3 ± 62.1	**0.012**
BMO-MRW _NI, μm	189.5 ± 60.5	220.0 ± 104.9	**0.012**	181.7 ± 68.1	188.8 ± 62.6	0.061
BMO-MRW _N, μm	170.5 ± 53.4	200.9 ± 82.5	**0.002**	158.4 ± 54.1	171.0 ± 55.8	**< 0.001**
BMO-MRW _NS, μm	151.8 ± 64.0	182.3 ± 79.9	**< 0.001**	164.0 ± 51.0	174.4 ± 47.5	**< 0.001**
RNFL_average, μm	66.1 ± 20.5	65.0 ± 19.5	0.334	58.0 ± 21.4	59.5 ± 19.8	0.410
RNFL_TS, μm	83.6 ± 40.4	78.3 ± 34.3	0.063	71.8 ± 33.6	69.3 ± 25.4	0.129
RNFL_T, μm	61.3 ± 27.5	61.1 ± 26.9	0.410	55.4 ± 23.4	57.7 ± 20.7	0.608
RNFL_TI, μm	76.3 ± 32.4	76.8 ± 37.9	0.473	67.7 ± 31.4	65.0 ± 31.2	0.694
RNFL_NI, μm	67.0 ± 31.2	65.7 ± 26.1	0.622	61.7 ± 23.5	64.3 ± 21.2	0.602
RNFL_N, μm	57.0 ± 22.3	54.3 ± 20.2	0.127	45.9 ± 22.9	49.7 ± 21.6	0.153
RNFL_NS, μm	70.5 ± 37.7	64.7 ± 30.0	0.069	65.6 ± 29.3	66.1 ± 29.9	0.886

*POAG* primary open-angle glaucoma, *XFG* exfoliation glaucoma, *IOP* intraocular pressure, *LCD* lamina cribrosa depth, *BMO-MRW* Bruch’s membrane opening-minimum rim width, *G* global, *TS* superotemporal, *T* temporal, *TI* inferotemporal, *NI* inferonasal, *N* nasal, *NS* superonasal, *RNFL* retinal nerve fiber layer. Data are presented as mean ± standard deviation. Statistically significant p-values are shown in bold.

When comparing the preoperative and postoperative LC depth, the average LC depth and most of the sectoral LC depths were significantly decreased after trabeculectomy in both the POAG and XFG groups. Similarly, the global and most sectoral BMO-MRW thicknesses increased significantly after trabeculectomy in both POAG and XFG eyes. However, the BMO area and RNFL thickness showed no significant difference before and after surgery in both groups (Table 2).

When factors associated with the degree of changes in LC depth after trabeculectomy were investigated, age, postoperative IOP, preoperative LC depth, and presence of XFG were found to be significant in univariable analysis. In multivariable analysis, lower postoperative IOP, deeper preoperative LC depth, and the absence of XFG remained significantly associated with the degree of changes in LC depth after trabeculectomy (Table 3). In the regression analysis to identify factors associated with increased BMO-MRW after trabeculectomy, lower postoperative IOP was significantly associated with increased BMO-MRW, but the presence of XFG was not (Table 4).

**Table 3:** Factors associated with the degree of lamina cribrosa shallowing (per 10 µm) after trabeculectomy.

	Univariable		Multivariable	
	Beta (95% CI)	p value	Beta (95% CI)	p value
Age, per 1-year older	− 0.23 (− 0.43, − 0.02)	**0.034**	− 0.08 (− 0.24, 0.09)	0.360
Gender, male	1.92 (− 0.81, 4.65)	0.173		
Presence of XFG	− 3.45 (− 5.92, − 0.99)	**0.008**	− 3.09 (− 5.06, − 1.11)	**0.003**
Diabetes	− 1.28 (− 4.77, 2.22)	0.477		
Hypertension	− 1.04 (− 4.12, 2.05)	0.513		
Axial length, mm	− 0.002 (− 1.11, 1.11)	0.997		
Central corneal thickness, 40 μm	− 0.4 (− 1.92, 1.08)	0.588		
Visual field MD, dB	0.11 (− 0.08, 0.30)	0.256		
Visual field PSD, dB	0.28 (− 0.15, 0.72)	0.206		
preoperative IOP, mmHg	− 0.02 (− 0.25, 0.21)	0.874		
postoperative IOP, mmHg	− 0.57 (− 1.00, − 0.14)	**0.012**	− 0.37 (− 0.71, − 0.03)	**0.037**
pre- and postooperative IOP difference, mmHg	0.05 (− 0.13, 0.23)	0.589		
preoperative LC depth, 10 μm	0.20 (0.13, 0.26)	**< 0.001**	0.16 (0.1, 0.23)	**< 0.001**
preoperative BMO-area, μm^2^	1.09 (− 1.60, 3.78)	0.431		
preoperative global BMO-MRW thickness, 10 μm	0.02 (− 0.30, 0.34)	0.889		
preoperative average RNFL thickness, 10 μm	0.13 (− 0.51, 0.78)	0.683		

*XFG* exfoliation glaucoma, *MD* mean deviation, *PSD* pattern standard deviation, *dB* decibel, *IOP* intraocular pressure, *LC* lamina cribrosa, *BMO-MRW* Bruch’s membrane opening-minimum rim width, *RNFL* retinal nerve fiber layer. Beta coefficients were calculated based on the 10 µm decrease in the lamina cribrosa depth. Statistically significant p-values are shown in bold.

**Table 4:** Factors associated with increase in the global Bruch’s membrane opening minimum rim width thickness (per 10 µm) after trabeculectomy.

	Univariable		Multivariable	
	Beta (95% CI)	p value	Beta (95% CI)	p value
Age, per 1-year older	− 0.22 (− 0.46, 0.02)	0.081	− 0.19 (− 0.43, 0.05)	0.12
Gender, male	1.18 (− 2.08, 4.44)	0.480		
Presence of XFG	− 2.02 (− 5.06, 1.02)	0.199		
Diabetes	0.85 (− 3.28, 4.98)	0.688		
Hypertension	1.83 (− 1.78, 5.45)	0.324		
Axial length, mm	0.66 (− 0.67, 1.99)	0.337		
Central corneal thickness, 40 μm	− 1.12 (− 2.88, 0.64)	0.222		
Visual field MD, dB	0.22 (− 0.003, 0.43)	0.058	0.15 (− 0.06, 0.37)	0.160
Visual field PSD, dB	0.48 (− 0.02, 0.98)	0.065		
preoperative IOP, mmHg	0.05 (− 0.22, 0.33)	0.699		
postoperative IOP, mmHg	− 0.64 (− 1.15, − 0.13)	**0.016**	− 0.56 (− 1.07, − 0.06)	**0.033**
pre- and postooperative IOP difference, mmHg	0.13 (− 0.07, 0.34)	0.211		
preoperative LC depth, 10 μm	0.03 (− 0.06, 0.13)	0.493		
preoperative BMO-area, μm^2^	− 2.71 (− 5.82, 0.40)	0.093	− 3.06 (− 5.95, − 0.16)	**0.043**
preoperative global BMO-MRW thickness, 10 μm	0.28 (− 0.05, 0.62)	0.104		
preoperative average RNFL thickness, 10 μm	0.55 (− 0.19, 1.30)	0.152		

*XFG* exfoliation glaucoma, *MD* mean deviation, *PSD* pattern standard deviation, *dB* decibel, *IOP* intraocular pressure, *LC* lamina cribrosa, *BMO-MRW* Bruch’s membrane opening-minimum rim width, *RNFL* retinal nerve fiber layer. Beta coefficients were calculated based on the 10 µm increase in the BMO-MRW. Statistically significant p-values are shown in bold.

**Figure 1:**
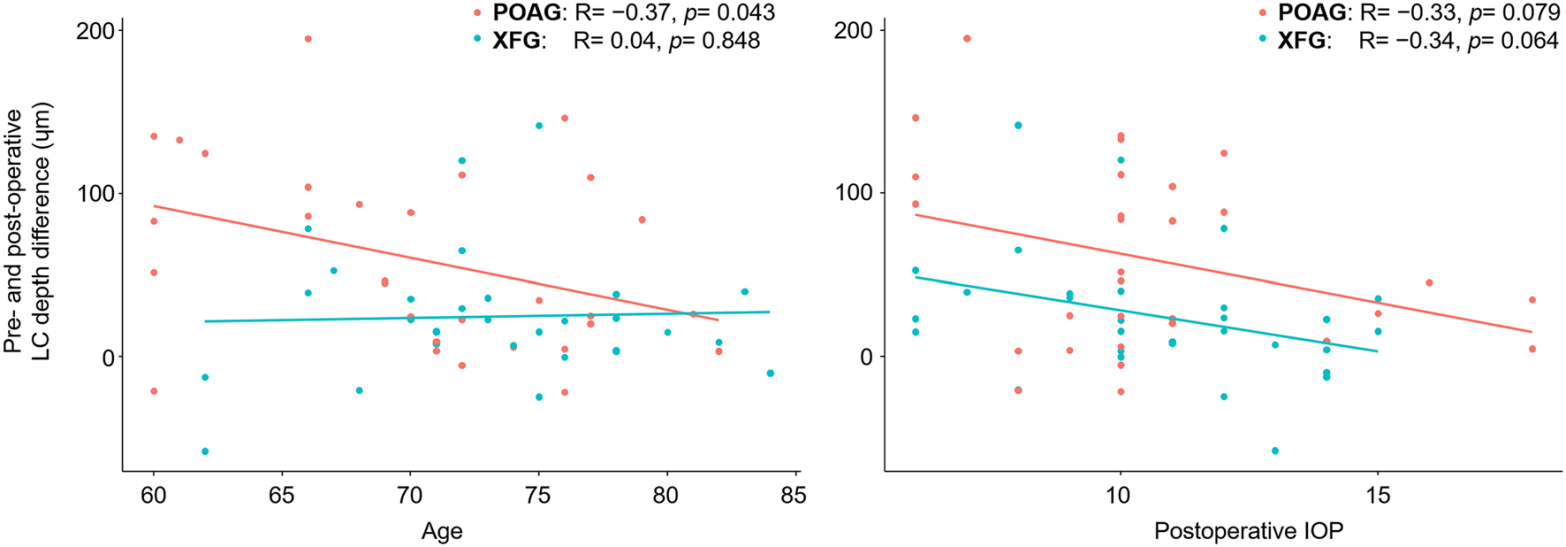
Left panel—Correlation plots between pre- and postoperative lamina cribrosa (LC) depth difference and age in eyes with primary open-angle glaucoma (POAG) and those with exfoliation glaucoma (XFG). Right panel—Correlation between pre- and postoperative LC depth difference and postoperative intraocular pressure (IOP) in POAG and XFG eyes. R: Pearson’s correlation coefficients.

Correlation analysis showed that age was significantly correlated with the degree of changes in LC depth after trabeculectomy in POAG eyes, but this correlation was not significant in eyes with XFG (Fig. 1). Postoperative IOP was significantly correlated with LC depth changes both in POAG and XFG eyes (Fig. 1).

**Figure 2:**
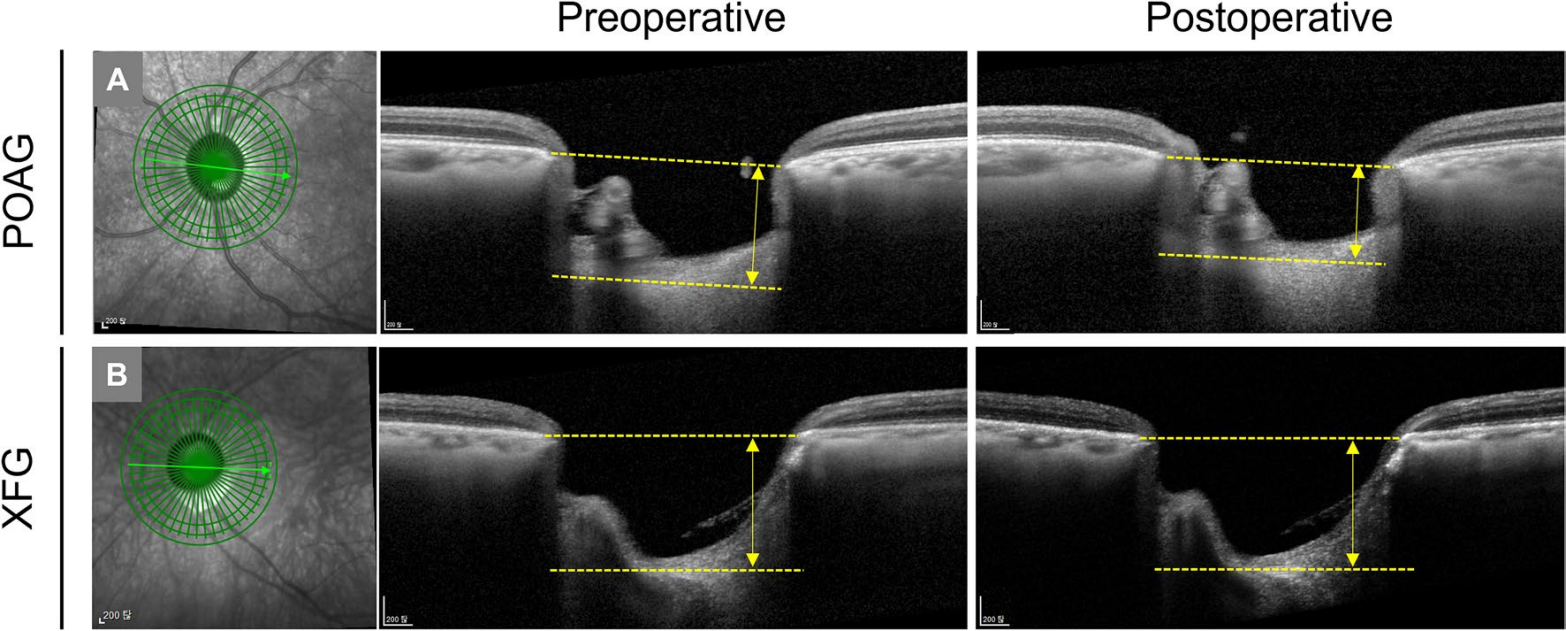
Representative cases of pre- and post-operative lamina cribrosa (LC) depth changes in POAG (**A**) and XFG (**B**) eyes. (**A**) 77-year-old male. Axial length (AL) 25.1 mm. Visual field mean deviation (MD) − 21.9 dB. Significant LC depth shallowing is observed from 775 µm preoperatively (IOP 27 mmHg) to 665 µm postoperatively (6 months. IOP 10 mmHg). (**B**) 76-year-old male. AL 24.3 mm. MD − 22.4 dB. LC depth is 911 µm preoperatively (IOP 27 mmHg) and 889 µm postoperatively (6 months. IOP 11 mmHg).

Representative cases of LC depth changes after trabeculectomy in POAG and XFG eyes are presented in Fig. 2.

## Discussion

In this study, we confirmed that the LC depth becomes shallow in the ONH of both POAG and XFG eyes in response to decreased IOP after trabeculectomy. In particular, the degree of change in LC depth was significantly lower in XFG eyes than in POAG eyes. These results suggest that the properties of the connective tissue of the LC may differ between POAG and XFG, which may lead to differences in the degree of LC response to IOP lowering after trabeculectomy.

The LC is a trabecular structure composed of connective tissue that supports RGC axons passing through the pores^2^. Therefore, the properties and amount of connective tissue can be critical factors to maintain axon support. The connective tissue constituting the LC has been reported to have particular structures and properties in XFG^10^. Specifically, elastic fibers in the LC of XFS eyes show a disorganized and fragmented appearance^10^. In addition, the expression of elastic proteins, such as elastin and fibrillin-1, and the elastin crosslinking protein LOXL1 was reported to be downregulated in the LC of XFS^16^. Regarding the deficient connective tissue characteristics of LCs in XFG eyes, it is believed that the LC can be more deformed by IOP and is more vulnerable to pressure-induced axonal damage^16^, which is supported by the findings of recent studies in which the ONH of XFG eyes had a thinner LC with greater curvature than that of POAG eyes with similar glaucomatous damage^11,12^.

In this study, we focused more on how particular properties of the LC in eyes with XFG may affect the response to decreased IOP after trabeculectomy. Since the preoperative LC depth was similar between the POAG and XFG eyes included in the present study, the degree of preoperative LC deformation was also considered to be similar between the two groups. Interestingly, however, the amount of recovery of the LC depth in response to the IOP decrease after trabeculectomy was smaller in XFG eyes than in POAG eyes. Based on these findings, we speculated that, due to abnormalities in the connective tissue and ECM, XFG eyes experience not only passive deformation of the LC related to IOP elevation, but also more severe active remodeling, which is an irreversible process following the deformation. Due to the irreversible properties of the LC in XFG eyes, the degree to which the LC becomes shallow in response to IOP reduction after trabeculectomy may also be decreased. In fact, the ECM not only mechanically supports the structure of the surrounding cells but also regulates cellular homeostasis and signal transduction through various pathways^17,18^. For example, integrins bound to the ECM mediate various biological processes, such as contractility of the surrounding cells and production and deposition of matrix proteins^19^. In other words, defective elastic fibers in the LC in XFG eyes may also affect the cells and ECM in the LC which determines the vulnerability to the deformation or reversibility of the LC. How the altered properties of connective tissues and ECM, especially elastic fibers and elastin, in the LC of XFG eyes affect the interaction of the ECM with cells and tissues composing the LC should be elucidated in the future.

The BMO-MRW is an OCT-based quantitative measurement of the NRR of the ONH^20,21^. Similar to the phenomenon in which the LC depth becomes shallow with the decreased IOP after trabeculectomy, the BMO-MRW also thickens in response to decreased IOP following trabeculectomy^3,7,22^. However, unlike the LC depth, the association between the increase in BMO-MRW after trabeculectomy and the presence of XFG was not significant in this study. We interpreted this finding to be due to the difference between the cellular and extracellular components constituting the BMO-MRW and the LC. It is known that the peripapillary RNFL, NRR, and LC have different types of cells and amounts of ECM surrounding the axons of the RGCs^2^. Hence, it is thought that the difference according to characteristics of the ECM observed in XFG eyes may also differ depending on the region of the ONH. To confirm these findings, analyses with more patients will be needed in the future.

Age is an important factor in determining the degree to which ONH structures, including the LC and NRR, respond to IOP. Several studies reported that younger age is associated with the degree of LC reversal or NRR thickening after trabeculectomy, which was also confirmed in this study^4,7^. However, this association was not significant when including patients with XFG. These results may be because we limited the patients’ age to those > 60 years to minimize the effect of the age difference between patients with POAG and XFG in this study. Consequently, the analysis included fewer younger individuals; therefore, the effect of age on the changes in the ONH according to postoperative IOP reduction may be less pronounced. Alternatively, these results could be explained by the fact that the correlation between age and the degree of changes in LC depth after trabeculectomy was different between POAG eyes and XFG eyes. In POAG eyes, as age increased, the degree of changes in LC depth following IOP reduction increased, but this correlation was not significant in XFG eyes. These findings also suggest that age- and IOP-sensitive characteristics of the ECM in the LC may be altered in XFG.

This study found that the postoperative LC shallowing was more closely related to postoperative IOP rather than to the difference between pre- and postoperative IOP. This finding, which is contrary to published literature, may be attributed to the larger proportion of XFG eyes in the present study than those in previous studies. Furthermore, in XFG eyes, the postoperative IOP may be more relevant to LC reversal, as the preoperative IOP measurement may not accurately reflect the greater IOP variability in XFG. An additional reason for this paradoxical finding may be that the postoperative IOP was measured regularly after surgery in each patient, but preoperative IOP measurements were not consistent for each patient in terms of frequency and number.

In this study, some cases demonstrated postoperative LC deepening rather than shallowing despite the IOP reduction after trabeculectomy. Similar findings were also reported previously, and old age and thick baseline RNFL were found to be the factors associated with deepening of LC after trabeculectomy^23^. In the present study, compared to the eyes with postoperative LC shallowing, eyes with LC deepening had a shallower preoperative LC and less response of BMO-MRW to IOP lowering after trabeculectomy. However, preoperative or postoperative IOP and baseline visual field MD were not significantly different between the two groups. Comparison with statistical analysis was not feasible due to the limited number of cases with LC deepening after trabeculectomy. We speculate that this finding may be related to the active process of connective tissue remodeling occurring in the LC, which may overcome the passive LC deformation by IOP reduction. Further study with a larger number of patients is needed to clarify the factors associated with this rare phenomenon.

This study has several limitations. First, the number of patients included in this study was small. For this reason, only the severity of glaucoma, and not the age, was matched between POAG and XFG eyes, which resulted in a marginal difference in the age between the two groups. However, age was adjusted later in the regression analysis, which confirmed that the LC response after trabeculectomy in XFG eyes was less than that in POAG eyes in an age-independent manner. Second, although the LC depth becomes shallow as the IOP decreases after trabeculectomy, it does not mean that the RNFL thickness increases or that the VF of the patients improves accordingly. Therefore, the clinical significance of the results of this study may be limited. However, the results of this study showing that the ONH in XFG eyes was more resistant to changes with the decrease in IOP may suggest that the ONH in XFG eyes is more likely to undergo irreversible remodeling, which can be one reason to imply that glaucoma progresses more rapidly in XFG eyes. As with RNFL or BMO-MRW, if LC-related parameters such as LC depth or thickness can be standardized and easily measured in the future, the results of this study may be better utilized, increasing their clinical relevance. Third, due to the retrospective study design, the intervals of OCT exams after trabeculectomy were different for each patient. However, by limiting the analysis period to 3–6 months after surgery, acute changes of the ONH occurring at the early stage after surgery were excluded from analysis. Fourth, when determining the pre- and postoperative IOP values, we did not specifically consider IOP fluctuation. There might have been more severe IOP fluctuations in the XFG eyes, especially prior to trabeculectomy^24^. Although we determined preoperative IOP as the average IOP measured one month before surgery, higher peak IOP, as observed in XFG eyes, may not have been reflected in the analysis. Fifth, we were unable to evaluate the anterior scleral canal opening (ASCO) and circumpapillary (cp)-choroidal thickness as confounding factors which could affect the LC depth^25–27^ owing to the absence of the enhanced depth imaging (EDI) data. However, we assumed that the ASCO and cp-choroidal thickness would show no significant inter-group difference given that AXL, which is strongly related to ASCO and cp-choroidal thickness^28,29^, were not different between the two groups. Finally, it was not possible to analyze whether or how fast the LC depth, which had become shallow after surgery, recovered over time. It was also not known how it differed between POAG and XFG eyes. This will be confirmed through a prospective study with more patients in the future.

In conclusion, we found that the phenomenon of LC depth shallowing with IOP reduction after glaucoma filtration surgery was less significant in patients with XFG compared to those with POAG. These findings suggest that eyes with XFG may have distinct connective tissue and ECM properties in the LC, which could be more resistant to IOP reduction and more likely to undergo irreversible remodeling.

## Methods

### Study subjects

For this retrospective interventional study, we analyzed the medical records of all patients who underwent trabeculectomy at Yeungnam University Hospital from March 2020 to August 2021 and selected patients who fulfilled the following inclusion and exclusion criteria. The study was approved by the Yeungnam University Hospital Institutional Review Board (IRB) and followed the tenets of the Declaration of Helsinki (IRB No. 2022-03-006). Considering the nature of the retrospective study, the need for informed consent was waived by the IRB of the Yeungnam University Hospital.

The inclusion criteria were as follows: (1) patients aged older than 60 years, (2) patients diagnosed with POAG or XFG that underwent trabeculectomy owing to uncontrolled IOP despite medical IOP lowering treatment or significant risk of glaucoma progression, (2) patients who underwent a spectral-domain OCT (SD-OCT) examination within 1 month before the trabeculectomy, (3) patients who underwent an SD-OCT examination between 3 and 6 months after trabeculectomy, and (4) patients with stable IOP below 20 mmHg after the time of trabeculectomy to postoperative SD-OCT examination. All patients underwent a limbal- or fornix-based trabeculectomy with mitomycin C, performed by a glaucoma specialist (DYP, SCC).

Patients with a history of intraocular surgery except for uncomplicated cataract surgery were excluded. Cases requiring additional procedures because of hypotony or increased IOP after surgery, or cases with IOP less than 6 mmHg were also excluded.

POAG was defined as when the following criteria are satisfied: (1) the presence of characteristic glaucomatous optic disc appearances (increased cupping, focal or diffuse loss of NRR, or RNFL defect) with corresponding VF defect, (2) open angle on gonioscopic evaluation, (3) untreated IOP greater than 21 mmHg, and (4) no conditions causing secondary IOP elevation^30^.

XFG was diagnosed when criteria (1), (2), and (3) were satisfied together with the presence of exfoliation materials observed at the anterior lens capsule and/or at the pupillary border^30^.

We collected the following information from the subjects included in this study: age, sex, presence of hypertension or diabetes, phakic status, average IOP under IOP-lowering medical treatment within 1 month before surgery, average IOP between 3 and 6 months after surgery, AXL, CCT, vertical cup to disc ratio, disc size, VF mean deviation (MD), pattern standard deviation, VF index, and SD-OCT measurements (BMO and RNFL parameters) taken before and after surgery. The time interval from the surgery to postoperative SD-OCT measurement was also collected.

We matched eyes with POAG eyes to eyes with XFG using VF MD (< 2 dB) to minimize and control the effect of severity of glaucoma on changes in LC depth and BMO minimum rim width (BMO-MRW) after trabeculectomy.

### Measurement of BMO-MRW and LC depth using SD-OCT

**Figure 3:**
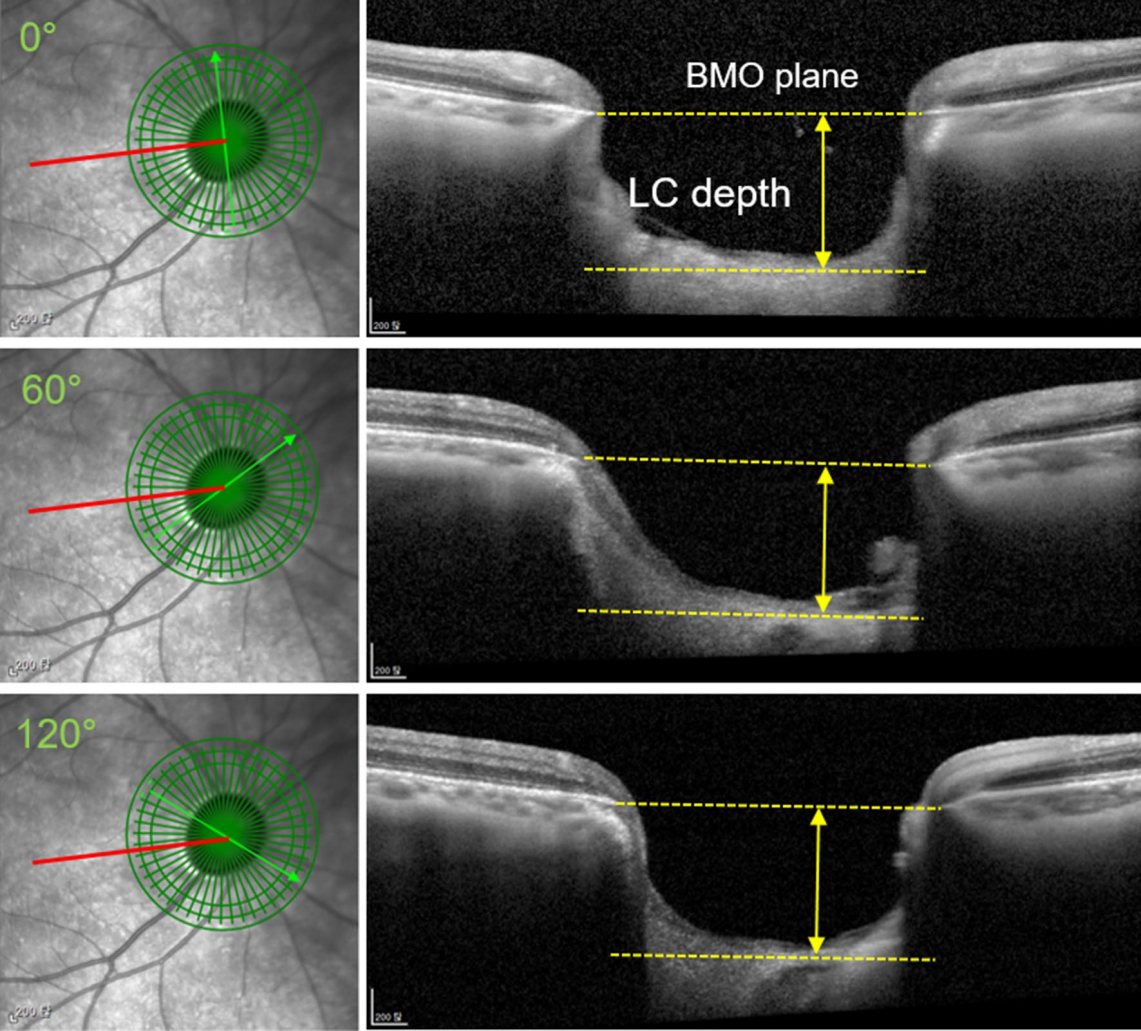
Measurement of lamina cribrosa (LC) depth using optical coherence tomography. The FoBMO axis (red line) was defined as the line connecting the center of the Bruch’s membrane opening (BMO) and fovea. A vertical line to the FoBMO axis passing through the center of the BMO was defined as 0°, and the LC depth was measured manually at 0°, 60°, and 120° clockwise in the right eye. LC depth (yellow solid line) was defined as the maximum perpendicular distance between the plane connecting both BMOs and the anterior surface of the LC (yellow dotted lines).

Spectralis SD-OCT (Heidelberg Engineering, Heidelberg, Germany) was used to measure BMO-related parameters and LC depth in the ONH. Scans were focused on the center of the BMO, and 24 radial equidistant cross-sectional images were obtained. The BMO-MRW and RNFL thickness values were obtained on a global and sectoral basis using the device’s standard operating software (Heidelberg Eye Explorer, version 1.10.2.0; Heidelberg Engineering) The BMO area was also presented automatically in the software. If necessary, we manually corrected the automated delineation of the internal limiting membrane and BMO. Preoperative measurements of LC depth were determined using the SD-OCT scans taken within a month before trabeculectomy, and postoperative measurements were taken between 3 and 6 months after surgery. To measure LC depth, we evaluated radial cross-sectional images of ONH at 3 locations with equal angular differences (60°). The line connecting the center of the BMO and fovea was defined as the FoBMO axis. A vertical line to the FoBMO axis passing through the center of the BMO was defined as 0°, and the LC depth was measured manually at 0°, 60°, and 120° clockwise in the right eye and counterclockwise in the left eye using a caliper tool in the software. LC depth was defined as the maximum perpendicular distance between the plane connecting both BMOs and the anterior surface of the LC. (Fig. 3) Measurement of LC depth was performed by 2 observers (SKS, DYP) who were blinded to the clinical information of the participants, and the average of the two measurements was used for the analyses.

### Statistical analyses

Intraobserver (two consecutive measurements by SKS) and interobserver (measured by SKS and DYP) reproducibility of measurements of the LC depth and BMO distances were assessed by calculating ICCs. OCT images of 20 randomly selected patients were used for this analysis. The Wilcoxon signed-rank test was performed to compare the variables before and after trabeculectomy. Univariable and multivariable regression analyses using a generalized linear model were performed to identify factors associated with the amount of changes in LC depth and BMO-MRW after trabeculectomy. Variables with a P-value < 0.1 in the univariable analysis were included in the multivariable analysis. Beta coefficients were calculated based on 10-µm changes in LC depth and BMO-MRW. Statistical significance was established at P < 0.05. Statistical analyses were performed using SPSS 24.0 (IBM, Armonk, NY) and R 3.5.3 (R Foundation for Statistical Computing, Vienna, Austria).

## Supplementary Information

Supplementary Table 1.

## Data Availability

The datasets analysed during the current study available from the corresponding author on reasonable request. The data are not publicly available due to privacy and ethical issues.
