# Supplementary material for: Comparison of lamina cribrosa depth shallowing after trabeculectomy between primary open-angle glaucoma and exfoliation glaucoma
Source: Sci Rep. 2022 Sep 20;12:15702. doi: 10.1038/s41598-022-19785-2 (PMC9489704; doi:10.1038/s41598-022-19785-2)
Supplement: Supplementary file 1 — Supplementary Table 1. [file 41598_2022_19785_MOESM1_ESM.docx]

**Supplementary** **Table 1. Intra- and inter-observer reproducibility in measurement of the lamina cribrosa depth**

| Parameters | Intraobserver ICC (95% CI) | Interobserver ICC (95% CI) |
| --- | --- | --- |
| Lamina cribrosa depth at 0°, µm | 0.942 (0.901–0.982) | 0.954 (0.919–0.988) |
| Lamina cribrosa depth at 60°, µm | 0.924 (0.852–0.988) | 0.962 (0.925–0.994) |
| Lamina cribrosa depth at 120°, µm | 0.930 (0.883–0.979) | 0.937 (0.890–0.990) |

ICC: intraclass correlation coefficients
